# Synthesis of imine bond containing insoluble polymeric ligand and its transition metal complexes, structural characterization and catalytic activity on esterification reaction

**DOI:** 10.1080/15685551.2017.1332139

**Published:** 2017-05-28

**Authors:** İlyas Gönül, Burak Ay, Serkan Karaca, Oguz Yunus Saribiyik, Emel Yildiz, Selahattin Serin

**Affiliations:** ^a^ Arts and Science Faculty, Department of Chemistry, Çukurova University, Adana, Turkey; ^b^ Faculty of Engineering and Natural Science, Department of Genetic and Bioengineering, Gumushane University, Gumushane, Turkey

**Keywords:** Insoluble polymer, heterogeneous catalyst, esterification reaction, schiff base

## Abstract

In this study, synthesis of insoluble polymeric ligand **(L)** and its transition metal complexes [Cu(L)Cl_2_]·2H_2_O **(1)**, [Co(L)Cl_2_(H_2_O)_2_] **(2)** and [Ni(L)Cl_2_(H_2_O)_2_] **(3)**, having the azomethine groups, were synthesized by the condensation reactions of the diamines and dialdehydes. The structural properties were characterized by the analytical and spectroscopic methods using by elemental analysis, Fourier Transform Infrared, Thermo Gravimetric Analysis, Powder X-ray Diffraction, magnetic susceptibility and Inductively Coupled Plasma. The solubilities of the synthesized polymeric materials were also investigated and found as insoluble some organic and inorganic solvents. Additionally, their catalytic performance was carried out for the esterification reaction of acetic acid and butyl acetate. The highest conversion rate is 75.75% by using catalyst **1**. The esterification of butanol gave butyl acetate with 100% selectivity.

## 1. Introduction

**Scheme 1.**
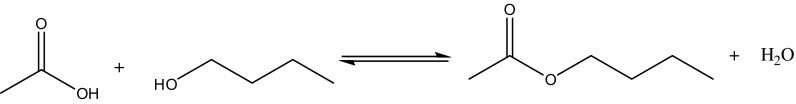
Synthesis of butyl acetate.

Poly(azomethine)s, known as polyimines or Schiff base polymers [1] are obtained by derivating from different diamines and dialdehydes [2]. The presence of the nitrogen atom in their backbone makes them environmentally stable [3]. Polymers are described as high molecular weight molecules formed by the recurrence of monomeric units addicted with covalent bonds [4]. Among many ligands, polyazomethine polymers have attracted great interest due to their different properties such as conjugated backbone and good thermal stability [4], imine sites [5], ability to form metal chelates [6] and mechanical strength [7]. Transition metal complexes of polyazomethine polymers, prepared by the reaction of metal salts and polymers containing electron donor groups like –CH=N–, are showed high catalytic activities in various chemical reactions such as oxidation [8], epoxidation of olefins [9,10] and esterification reactions. However, heterogeneous catalysts indicate higher catalytic activity [11,12] and enantioselectivity [13] in comparison to homogeneous catalysts [14]. N-butyl acetate is an important compound in the chemical industry (Scheme 1). Primarily, it is used in paint, coating manufacture, and lacquer industry. Because of its lower impact on the environment, n-butyl acetate is able to replace the toxic and teratogenic effect of ethoxy ethyl acetate that is often used as a solvent [15,16].

In this study, we present synthesis and characterization of an insoluble polymeric ligand **(L)** and its metal complexes [Cu(L)Cl_2_]·2H_2_O **(1)**, [Co(L)Cl_2_(H_2_O)(CH_3_OH)] **(2)** and [Ni(L)Cl_2_(H_2_O)_2_] **(3)**. The synthesized compounds were characterized by elemental analysis, Fourier Transform Infrared (FT-IR), Thermo Gravimetric Analysis (TGA), Inductively Coupled Plasma (ICP), Powder X-ray Diffraction (PXRD) analysis and magnetic susceptibility measurements. Their catalytic activities were investigated on the esterification reaction.

## 2. Experimental

### 2.1. Materials and methods

CuCl_2_·2H_2_O, CoCl_2_·6H_2_O, NiCl_2_·6H_2_O, ethanol, 2,4 diamino toluene, glutaraldehyde (25% water solution), n-butanol, glacial acetic acid, n-butyl acetate, dichloromethane were purchased from Sigma Aldrich. pH values of the reactions were measured using the Hanna 211 pH meter. Infrared spectra was obtained on a Perkin-Elmer RX-1 (KBr disk; 4000–400 cm^−1^) FT-IR spectrometer. TGA was performed with a Perkin Elmer Pyris Diamond TG/DTA N_2_ (50–800 °C range) at a heating rate of 10 °C/min. The characterization, selectivity % and catalytic conversion % studies of the esterification product, butyl acetate were analyzed by Thermo GC-FID. GC was performed using a Thermo GC-FID detector. A Perkin-Elmer Optima 2100 DV ICP-OES instrument was used for the ICP analysis. A Rigaku Miniflex system with CuKα radiation (*λ* = 1.54059 Å) was used for the PXRD studies. Magnetic susceptibility measurements were performed by using Sherwood Scientific magnetic susceptibility.

### 2.2. Synthesis of polymeric schiff base ligand

**Figure 1.**
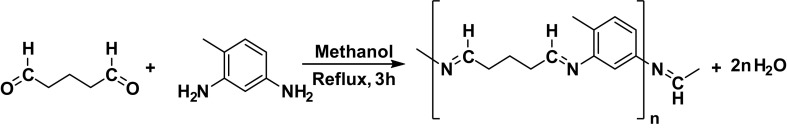
The structure of the synthesized polymeric Schiff base ligand.

The polymeric Schiff base ligand were prepared by refluxing 2,4-diamino toluene (1.222 g, 10 mmol) with corresponding glutaraldehyde (4.005 g in water solution 25%, 10 mmol) in methanol (50 mL) for 3 h. The dark brown solution was filtered, and insoluble solid was washed 2 times with 20 mL methanol. It was dried under vacuum and reaction yield was determined as 92.6% (Figure 1). Color: Dark Brown. M.P.: >300 °C. Anal. Calcd. For C_12_H_14_N_2_: C, 76.55; H, 8.57; N, 14.88. Found: C, 75. 81; H, 7.93; N, 15.18%. IR data (KBr pellet, cm^−1^): FT-IR (KBr pellet, cm^−1^): 3344–3213 *υ*(O–H)aromatic or water, 2928 *υ*(C–H)aliphatic, 1615 *υ*(CH=N), 1505 *υ*(C–C)aromatic [17].

### 2.3. Synthesis of polymeric transition metal complexes

**Figure 2.**
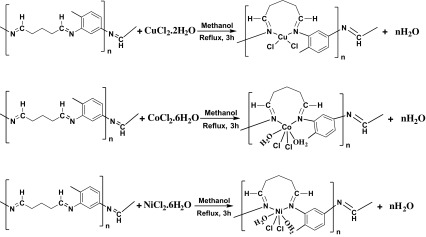
The structure of the synthesized polymeric coordination polymers.

The polymeric metal complexes were prepared as follows: It was first dissolved 2,4-diamino toluene (1.222 g, 10 mmol) in methanol (30 mL), and followed by addition of metal salts (0.170 g, 1.0 mmol) for CuCl_2_·2H_2_O; (0.238 g, 1.0 mmol) for CoCl_2_·6H_2_O; (0.237 g, 1.0 mmol) for NiCl_2_·6H_2_O; in CH_3_OH (20 mL). This reaction mixture was heated under reflux for 1 h and then glutaraldehyde was added (4.005 g from water solution 25%, 10 mmol) in the reaction medium and it was boiled for three hours. All of the polymeric metal complexes were collected by filtration, washed with MeOH and dried under vacuum. The synthesized compounds were obtained as powder (Figure 2) [17].

[Cu(L)Cl_2_]·2H_2_O **(1)**: Yield: 90.6%, Color: Light Brown. M.P.: >300 °C. Anal. Calcd. For C_12_H_19_Cl_2_N_2_Cu: C, 40.29; H, 5.35; Cl, 19.82; Cu, 17.76; N, 7.83%; found: C, 40.14; H, 4.94; Cl, 19.56; Cu, 15.69; N, 7.51%. FT-IR (KBr pellet, cm^−1^): 3442 and 3339 *υ*(O–H)water, 2950 *υ*(C–H)aliphatic, 1616 *υ*(CH=N), 1527 *υ*(C–C)aromatic, 584 *υ*(Cu–N).

[Co(L)Cl_2_(H_2_O)_2_] **(2)**: Yield: 92.3%, Color: Dark brown. M.P.: >300 °C. Anal. Calcd. For C_12_H_15_Cl_2_N_2_Co: C, 42.53; H, 5.77; Cl, 19.31; Co, 16.05; N, 7.63%; found: C, 42.24; H, 5.05; Cl, 18.94; Co, 15.12; N, 7.97%. FT-IR (KBr pellet, cm^−1^): 3332 and 3222 *υ*(O–H)water, 2942 *υ*(C–H)aliphatic, 1634 *υ*(CH=N), 1505 *υ*(C–C) aromatic, 699 *υ*(Co–N).

[Ni(L)Cl_2_(H_2_O)_2_] **(3)**: Yield: 91,4%, Color: Brown. M.P.: >300 °C. Anal. Calcd. For C_12_H_15_Cl_2_N_2_Ni: C, 40.84; H, 5.43; Cl, 20.09; Ni, 16.63; N, 7.94%; found: C, 40.55; H, 5.03; Cl, 19.76; Ni, 16.25; N, 7.85%. FT-IR (KBr pellet, cm^−1^): 3327–3215 *υ*(O–H) water, 2930, 2832 *υ*(C–H)aliphatic, 1633 *υ*(CH=N), 1505 *υ*(C–C)aromatic, 599 *υ*(Ni–N).

### 2.4. Esterification of butanol with acetic acid

The obtained butyl acetate was carried out at 25, 80 °C and boiling temperature in a three-necked flask (100 mL) equipped with a magnetic stirrer, a reflux condenser and a temperature controller in an oil bath. Butanol (4.95 g, 66.78 mmol) and acetic acid (4.083 g, 67.99 mmol) were added successively into the flask. After heating, the mixture is to be 25–80 °C-boiling temperature, the catalyst (0.05–0.10 g) was added to the mixture to initiate the reaction. The reaction mixture was stirred continuously for 4, 8 and 12 h. The products of the esterification reactions were collected at different time intervals, and identified by GC-FID.

## 3. Results and discussion

The metal compounds were synthesized according to appropriate methods. For the characterization of obtained the polyimine ligand and its metal complexes were characterized by melting point, elemental analysis, TGA, FT-IR, ICP, XRD analysis, and magnetic susceptibility. The synthesized compounds were insoluble so they can be used as heterogenous catalysts. Resolution of catalyst **1** is shown in Table 1.

**Table 1.** The physical and analytical properties of the synthesized compounds.

Compound	Yield %	M.P. °C	% Metal		Elemental analysis (% Cal./found)		
			*Cal.	Found	C	H	N
**Ligand**	92.6	>300	–	–	76.55/75.81	8.57/7.93	14.88/15.18
**1**	90.6	>300	17.76	15.69	40.29/40.14	5.35/4.94	7.83/7.51
**2**	92.3	>300	16.05	15.12	42.53/42.24	5.77/5.05	7.63/7.97
**3**	91.4	>300	16.63	16.25	40.84/40.55	5.43/5.03	7.94/7.85

* Cal: Calculated.

### 3.1. IR spectra

A strong and broad absorption band at the 1615 cm^−1^ shown by the synthesized polymeric ligand is attributable to the *υ*(C=N) stretching. In the complexes, this band shifted between 1616–1634 cm^−1^ indicating the coordination of azomethine nitrogen to the metal. This is supported by the appearance of new band in the region 584–699 cm^−1^ due to metal ligand bonding. The absorption bands relative to *υ*(O–H) stretching bands in the range of 3442–3213 cm^−1^ is shown the coordination of water to metal for the metal complexes [18]. The IR spectra are given in Table 2 and Figures S1–S4.

**Table 2.** IR spectral bands (cm^−1^) and magnetic susceptibility values of the compounds.

Compound	*υ*(OH)	*υ*(C=N)	*υ*(M–N)	*M*_eff_ (B.M.)	*n*
**L**	3344–3213	1615	–	–	–
**1**	3442–3339	1616	584	2.10	1
**2**	3332–3222	1634	699	4.41	3
**3**	3327–3215	1633	599	2.85	2

### 3.2. PXRD studies

**Figure 3.**
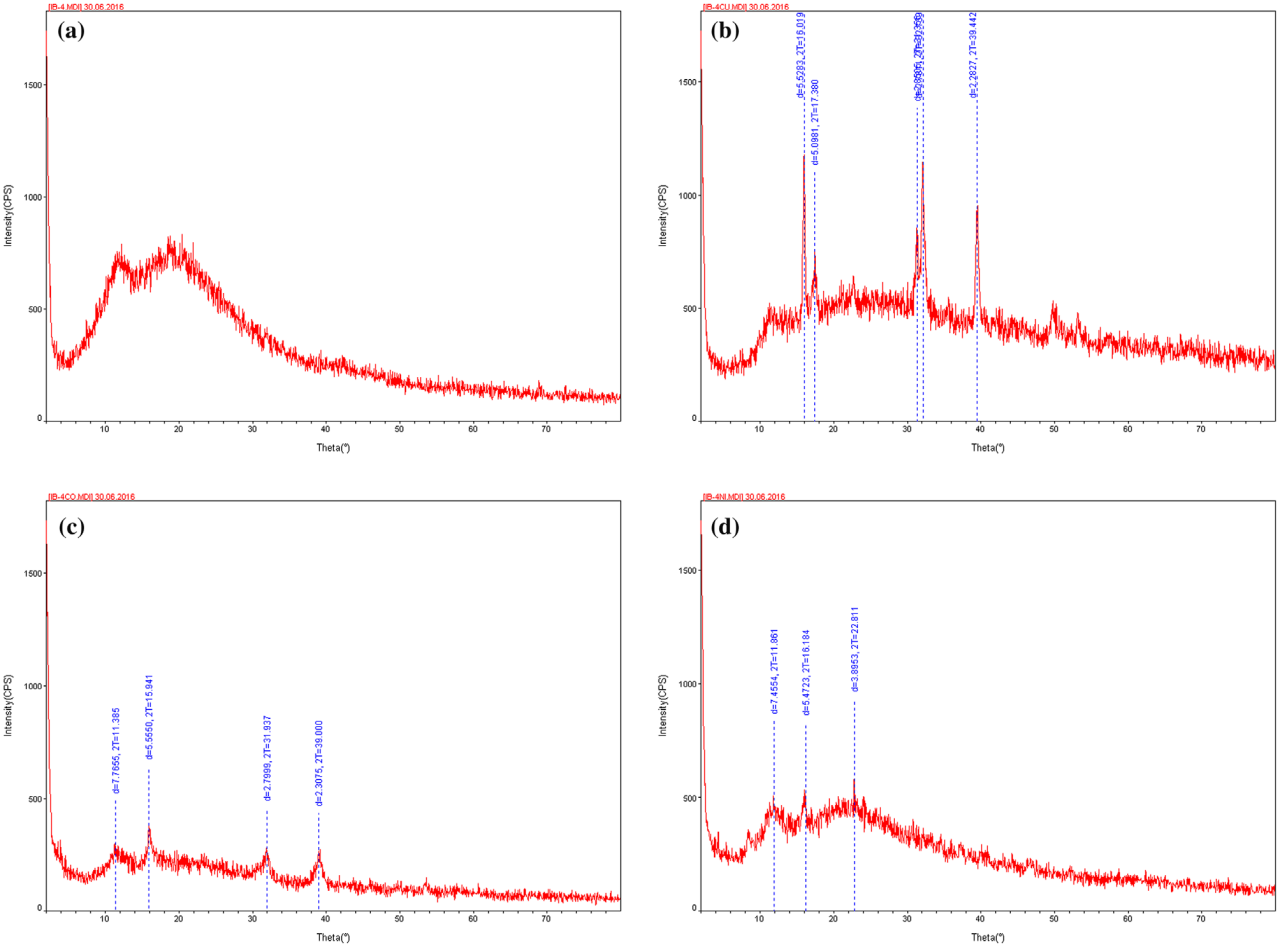
The XRD pattern of polymeric ligand **(a)** and its **1**-**(b)**, **2**-**(c)** and **3**-**(d)** complexes.

Powder diffraction data 2*θ* values of the complexes are shown in Figure 3 and Table 3. The powder XRD analyses of the compounds have been performed in order to determine the information of non-polycrystalline material because it couldn’t be obtained suitable crystals for single crystal X-ray analysis. Therefore the powder XRD analysis of metal complexes was found to be amorphous in nature instead of crystalline. The indexing and calculations of unit cell parameters were performed using Powder-X software and scattering angles (2*θ*) corresponding to the each reflection, inter-planar spacing (*d*) along with Miller’s indices and lattice constants were evaluated for complexes. The parameters like 2-Theta, *d*, BG, *I*, are, XS and FWHM are shown in Table 3. The FWHM is the Bragg diffraction angle (*b*) and *b* is the full width at half maximum. The density (*d*) of the complex was determined by the floatation method in a saturated solution of KBr, NaCl and benzene separately. The all values agree well with the suggested structure of the complexes.

**Table 3.** X-ray diffraction data of **1**–**3**.

2-Theta	*d*(A)	BG	Height	*I*%	Area	*I*%	FWHM	XS(A)
*Complex 1*								
16.019	5.5283	449	1176	100.0	251.3	100.0	0.277	311
17.380	5.0981	475	664	56.5	129.4	51.5	0.548	149
31.356	2.8505	596	834	70.9	73.6	29.3	0.247	365
32.159	2.7811	578	1141	97.0	247.3	98.4	0.351	245
39.442	2.2827	446	927	78.8	236.2	94.0	0.393	222
*Complex 2*								
11.385	7.7655	215	295	76.2	44.0	53.5	0.440	186
15.941	5.5550	231	387	100.0	70.7	86.0	0.363	230
31.937	2.7999	169	276	71.3	61.1	74.3	0.457	185
39.000	2.3075	140	262	67.7	82.2	100.0	0.539	159
*Complex 3*								
11.861	7.4554	403	504	89.5	42.3	57.8	0.335	250
16.184	5.4723	371	500	88.8	73.1	100.0	0.453	181
22.811	3.8953	452	563	100.0	25.1	34.4	0.181	537

### 3.3. Magnetic susceptibility of the complexes

The magnetic susceptibilities of the polymeric transition metal complexes were determined using the Evans balance at room temperature in the solid state. The magnetic correction for each complexes was estimated using Pascal’s constants. The magnetic moment data are shown in Table 2. According to the magnetic moment results, complex **1** has tetrahedral or square planar geometry; complexes **2** and **3** have octahedral geometries. The experimental *μ*_eff_ values for the polymeric complexes are within the range found for polymeric complexes d^9^ for [Cu(L)Cl_2_]·2H_2_O **(1)**, d^7^ for [Co(L)Cl_2_(H_2_O)_2_] **(2)** and d^8^ for [Ni(L)Cl_2_(H_2_O)_2_] **(3)** [19].

### 3.4. Thermal analysis studies

Thermogravimetric analysis (TGA) was carried out to examine the thermal stabilities of the polymeric complexes. TGA was performed under N_2_ atmosphere at 1 atm with a heating rate of 10 °C/min on a Perkin Elmer Diamond TGA/DTA. The objective of this section is to analyze the thermal behavior of the complexes having in view the composition confirmation, and evaluation of the crystal water molecules. TGA has been carried out at a heating rate of 20 °C per minute in the range of 20–900 °C under N_2_ atmosphere. On interpretation of the TGA curve, four distinct mass losses are observed and all curves are shown in Figures S5–S7. Polymeric [Cu(L)Cl_2_]·2H_2_O complex decomposition occurs in three steps. The first step is corresponding to the loss of a lattice water molecules in the temperature range 70–100 °C with a mass loss of 11.31% (Calcd: 11,13%). The second steps 100–900 °C are the decomposition of aromatic groups and the remaining organic substances with the mass loss 68.07% (Calcd: 67.82%), respectively. Mass loss, ended up in the 900 °C is form of CuO as the remaining mass 20.62% (Calcd: 21.05%). Polymeric [Co(L)Cl_2_(H_2_O)_2_)] [Co(L^1^)_2_] complex decomposition occurs in two steps. The first step is corresponding to the loss of a lattice water molecules in the temperature range 70–100 °C with a mass loss of 10.465% (Calcd: 10.75%). The second step 100–900 °C are the decomposition of aromatic groups and the remaining organic substances with the mass loss 68.81% (Calcd: 68.99%). After the 900 °C, the remaining weight of 20.73% is attributed to the final product of Co_2_O_3_ (Calcd: 20.26%). The thermogram of polymeric [Ni(L)Cl_2_(H_2_O)_2_] complex demonstrated weight loss in two steps. The first step is corresponding to the loss of a lattice water molecules in the temperature range 70–130 °C with a mass loss of 10.13% (Calcd: 10.20%). The second steps 130–710 °C are the decomposition of aromatic groups and the remaining organic substances with the mass loss 69.351% (Calcd: 70.24%), respectively. After the 710 °C, the remaining weight of 20.52% is attributed to the final product of NiO (Calcd: 19.56%) [19].

### 3.5. Molecular weight distribution of the polymers

It was performed solubility tests for synthesized polymers. As a result of the tests, we did not solved the structures in any organic and inorganic solvents (Table S1). So we could not performed GPC analysis to determination of the molecular weight of the polymers. After that, insoluble polymers were analysized with Quadrupole Time-of-flight Mass Spectrometry (Q-TOF MS) (maximum mass range: m/z: 17.000) and molecular weight of polymers could not be determined. This polymers have relatively high molecular weight, estimated to be in excess of 20.000 although the insolubility of the polymers have prevented exact determination [20].

### 3.6. Catalytic activity

Polymeric complexes have seldom been used as a heterogeneous catalyst for the esterification of butanol with acetic acid to butyl acetate [21]. There have been no reports on the esterification of butanol and acetic acid to butyl acetate using polyazomethine polymeric complexes, although butanol esterification reactions catalyzed by other types of the first row metal complexes in homogeneous and heterogeneous mediums [22]. In most of the cases, homogeneous acids and other metal complexes have been used for different catalytic conversion reactions. Homogeneous catalysis process has several disadvantages such as the product purification and reaction conditions. To avoid the loss of catalyst and for its recovery, many attempts have been taken, such as intercalating or encapsulating the metal complex into the layered compounds or within the cavities of a porous solid (e.g., zeolites), binding the metal complex to a polymeric matrix and employing the steric hindrance. Percent selectivity and characterization studies of the butyl acetate were carried out by using GC-FID. The esterification of butanol gave butyl acetate with 100% selectivity. The catalytic activity of the synthesized polymeric complexes was evaluated for the esterification of butanol in the presence of dichloromethane using a standard GC analysis technique. Table 4 summarizes the results, percentage of butanol conversion in the different reaction conditions. Conversion values were determined for butanol and butyl acetate calibration curves. To determine the performance of the catalyst, the yield butyl acetate formation from butanol and acetic acid was plotted as a function of different reaction conditions such as temperature, catalyst amount and reaction time. Although it was obtained low product conversion at room temperature, the selectivity was %100. When working boiling temperature 0.1 g polymeric Copper complex, catalyst performance was optimized giving the highest butyl acetate formation percentage of 75.75% after 12 h approximately 100% selectivity.

**Table 4.** % Conversion values under different conditions.

Reaction conditions			Complex 1	Complex 2	Complex 3
Reaction temperature (°C)	Catalyst amount (g)	Reaction time (h)	Conversion (%)	Conversion (%)	Conversion (%)
25	0.05	4	2.56	2.21	2.23
		8	3.11	2.91	2.91
		12	3.85	4.44	3.66
	0.10	4	2.65	2.32	2.46
		8	3.32	2.92	6.72
		12	4.01	4.42	3.78
80	0.05	4	16.52	15.25	21.03
		8	27.75	27.55	30.46
		12	35.37	36.41	37.61
	0.10	4	15.82	14.67	16.65
		8	26.62	25.39	24.37
		12	34.22	33.88	32.53
BT*	0.05	4	49.53	53.59	56.12
		8	57.49	65.20	63.37
		12	73.41	72.76	73.48
	0.10	4	49.46	50.68	53.50
		8	64.18	61.47	64.52
		12	**75.75**	**71.97**	**72.65**

* BT: Boiling temperature. The bold values represent the maximum conversion values.

To determine the catalytic conversion value without catalyst, one more control group experimental was performed. The esterification reaction was performed without catalyst and ~40% conversion value was observed as shown in the case of similar work [23]. Catalytic performance of the ligand was also done under same catalytic conditions. The GC/MS studies showed that 58% of conversion was observed using polymeric ligand as a catalyst. These studies supported that ligand is not showed high catalytic activity on esterification reaction as like synthesized heterogeneous catalysts.

After the catalytic studies, the catalysts filtered and washed with ether to avoid remained ester residues. Metal complexes were dried and TG analysis were performed secondly. After the TG analysis, no difference was observed in the thermal curves of the catalysts (Figures S8–S10). Although the loss of H_2_O was observed at 70–100 °C, it was observed that there was no change in the structure of the polymer in catalytic studies at boiling temperature. Since catalytic studies were carried out in solution medium, it is possible that water molecules separated at the end of the experiment were re-bound to the structure. The obtained results supported that there is no change in the lattice structure of water molecule in the polymeric structures.

### 3.7. Reaction mechanism for esterification of butyl acetate

**Figure 4.**
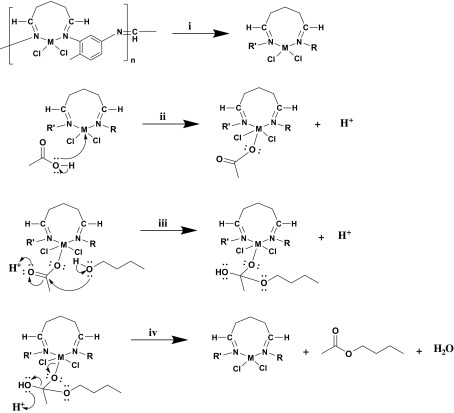
Proposed reaction mechanism for esterification of butyl acetate by using polymeric catalysts (M: Cu(II), Co(II) and Ni(II)).

The proposed mechanism of the esterification reaction is summarized in Figure 4. For easy description, the reproducible part of the synthesized polymer is abbreviated as *R* and *R*′ (i). When the catalytic activity of the ligand was examined, 58% conversion was obtained. The maximum yield of metal complexes was determined to be 75.75%. This result supported that the metal ion plays an active role in catalytic reaction, thus initiating the reaction with a proton separation by binding to the acetate ion (ii). Acetate ion attached to the complex structure is attached to the carbonyl carbon, and alcohol is bonded to the oxygen group, leaving the proton in the alcohol (iii). When the OH group in the final structure is separated by proton capture as H_2_O, ester formation is completed (iv). As the mechanism proceeds through the attachment of acetate ion to the metal, etheric structures is not form as by-products. This result was supported by GC-FID results. The polymeric catalysts synthesized for this reason were 100% selective in the esterification reaction. It is also believed that the chain structure of the polymeric catalyst helps the hydrophobic group of alcohol to approach the metal in position.

### 3.8. Separation and stability of catalyst

The major advantage of the use of heterogeneous catalysts is to recover the catalyst from the reaction mixture by simple filtration and recycle. The recovered heterogeneous catalysts can be reused in subsequent reactions. In order to assess the stability of the catalyst, the solid was filtrated at the end of the each reaction and washed with dichloromethane then dried at 50 °C. The dried catalyst was used under the same reaction conditions (butanol/acetic acid, 0.1 g polymeric catalysts, boiling temperature and 12 h). At the end of third reaction, butanol/acetic acid conversion did not change significantly. The recycling property is a very important parameter for a heterogeneous catalyst. This study showed that polymeric transition metal catalysts can be readily recycled more than three times (Table S2).

One of the best advantages of the insoluble polymeric structure as a catalyst could be accepted porosity of the surface of the catalysts. Insoluble low molecular weight catalyst has no 3D structure at the reaction condition therefore the expectation of that the rate of the reaction will be lower than the catalysts having 3D surface morphology. Another point we can concluded from our previous studies and TGA results, the polymeric insoluble catalysts is longer lifetime than the insoluble low molecular weight catalysts. Additionally, it is anticipated that durability of the 3D structure is much better than the monomers during the different reaction conditions.

## 4. Conclusions

We have successfully synthesized [Cu(L)Cl_2_]·2H_2_O **(1)**, [Co(L)Cl_2_(H_2_O)_2_] **(2)** and [Ni(L)Cl_2_(H_2_O)_2_] **(3)** as metal-organic catalytic from the reaction mixture of 2,4-diaminotoluene with CuCl_2_·2H_2_O, CoCl_2_·6H_2_O and NiCl_2_·6H_2_O metal salts were mixed and this reaction mixture prepared adding glutaraldehyde at reflux medium in methanol by classic method which is often result in stoichiometry (Dialdehyde:Diamine:Metal, 1:1:1). The synthesized polymeric compounds were characterized by using analytical and spectroscopic methods. The polymeric copper complex has higher catalytic activity than the Co(II) and Ni(II) transition metal complexes containing polyimine or polymeric Schiff base ligand as a heterogeneous catalyst. Because of the presence of the imine bridges, all the complexes are insoluble in hot/cold many common organic and inorganic solvents (Table S1). The solubility feature is important for the heterogeneous catalysts. Butyl acetate can be easily obtained by catalytic esterification of precursor by using butanol, acetic acid and catalysts. The esterification of butanol and acetic acid with 75.75% conversion gave butyl acetate with 100% selectivity. To the best of our knowledge, this is the first recorded on the esterification of butanol and acetic acid to butyl acetate by using complex **1** as a catalyst. In addition, catalytic conversions are increasing with increasing temperature but do not increase catalytic conversions with the amount of increased catalyst.

## Disclosure statement

No potential conflict of interest was reported by the authors.

## Supplemental data

The supplemental data for this article is available online at https://doi.org/10.1080/15685551.2017.1332139.

## Supplementary Material

TDMP_1332139_Supporting_Information.doc
