# Supplementary material for: Synthesis of imine bond containing insoluble polymeric ligand and its transition metal complexes, structural characterization and catalytic activity on esterification reaction
Source: Des Monomers Polym. 2017 May 28;20(1):441–8. doi: 10.1080/15685551.2017.1332139 (PMC5784868; doi:10.1080/15685551.2017.1332139)
Supplement: TDMP_1332139_Supporting_Information.doc [file TDMP_A_1332139_SM7391.doc]

# SUPPORTING INFORMATION

# Synthesis of Imine Bond Containing Insoluble Polymeric Ligand and its Transition Metal Complexes, Structural Characterization and Catalytic Activity on Esterification Reaction

İlyas Gönül ^a^, Burak Ay ^a^, Serkan Karaca ^a^, Oğuz Yunus Sarıbıyık ^b^, Emel Yildiz ^a^, Selahattin Serin ^a^ ^a^ *Çukurova University, Arts and Science Faculty, Department of Chemistry, 01330, Adana, Turkey ^b^ Department of Genetic and Bioengineering, Faculty of Engineering and Natural Science, Gumushane University, Gumushane, Turkey*

[*ilyasgonul01@gmail.com*](mailto:ilyasgonul01@gmail.com)

**Figure S1.** IR spectrum of polymeric schiff base ligand (L).

**Figure S2.** IR spectrum of polymeric complex 1.

**Figure S3.** IR spectrum of polymeric complex 2.

**Figure S4.** IR spectrum of polymeric complex 3.


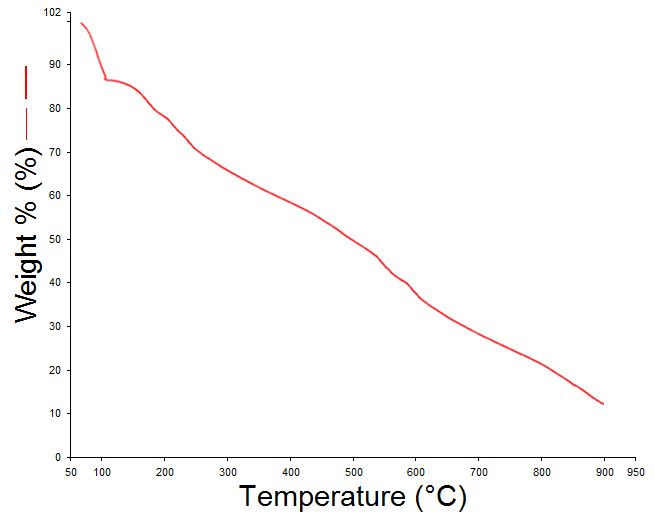


**Figure S5.** TGA curve of the polymeric complex 1.


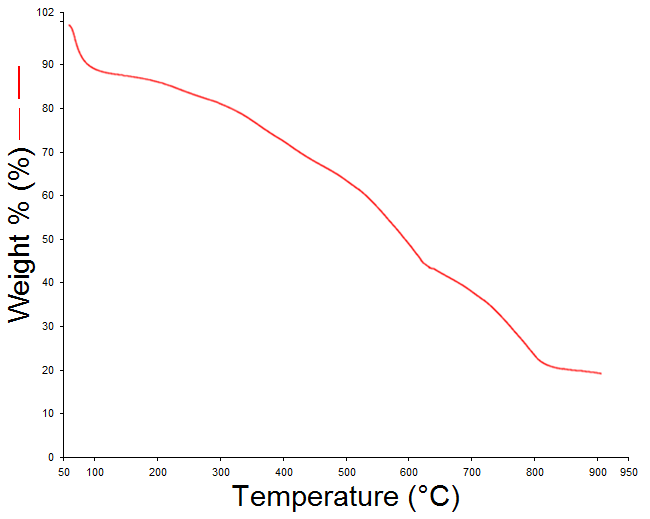


**Figure S6.** TGA curve of the polymeric complex 2.


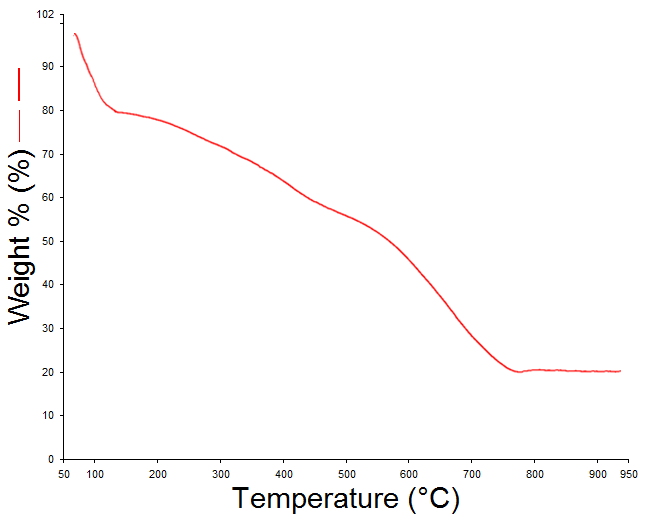


**Figure S7.** TGA curve of the polymeric complex 3.


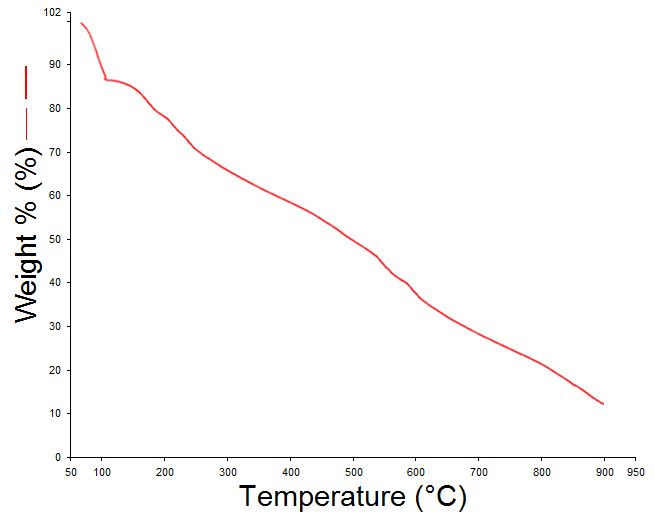


**Figure S8.** TGA curve of the polymeric complex 1 after catalytic studies.


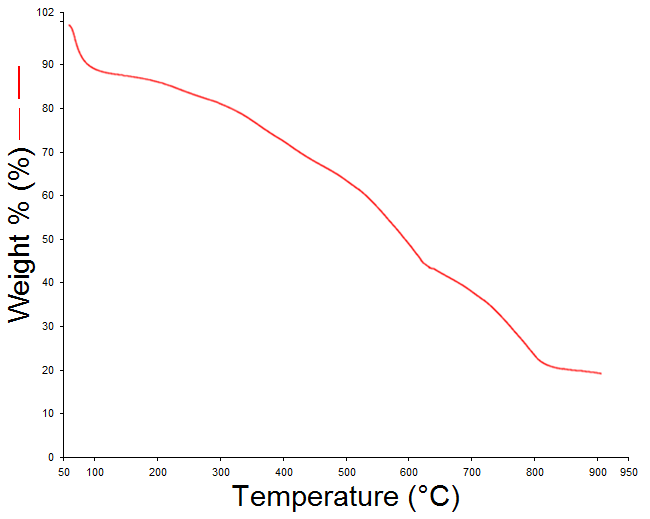


**Figure S9.** TGA curve of the polymeric complex 2 after catalytic studies.


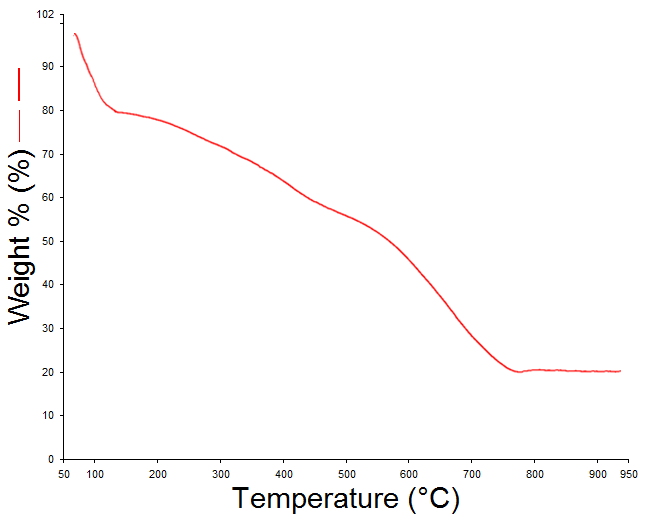


**Figure S10.** TGA curve of the polymeric complex 3 after catalytic studies.

**Table S1.** Solubility of the polymeric ligand and its metal complexes.

X : Insoluble

| _____________________________________________________ | | | | |
| --- | --- | --- | --- | --- |
| **Solvent** | **L** | **1** | **2** | **3** |
| _____________________________________________________ | | | | |
| Distilled water | X | X | X | X |
| DMF | X | X | X | X |
| DMSO | X | X | X | X |
| CH_3_OH | X | X | X | X |
| CH_3_CH_2_OH | X | X | X | X |
| Chloroform | X | X | X | X |
| Acetone | X | X | X | X |
| THF | X | X | X | X |
| Acetonitrile | X | X | X | X |
| Toluene | X | X | X | X |
| Hexane | X | X | X | X |
| Benzene | X | X | X | X |
| Diethyl ether | X | X | X | X |
| Ethyl acetate | X | X | X | X |
| Pentane | X | X | X | X |
| Xylene | X | X | X | X |
| Pyridine | X | X | X | X |
| 2-propanol | X | X | X | X |
| Petroleum ether | X | X | X | X |
| Heptane | X | X | X | X |
| Ethylene glycol | X | X | X | X |
| Chlorobenzene | X | X | X | X |
| Triethyl amine | X | X | X | X |
| _____________________________________________________ | | | | |

**Table S2**.

The stability of the polymeric copper catalyst

| __________________________________________ | |
| --- | --- |
| **Number of**  **Recycle** | **Butyl acetate Conversion (%)** |
|  | **Butanol/Acetic Acid** |
| __________________________________________ | |
| First reuse | 75.75  75.43  75.20 |
| Second reuse |  |
| Third reuse |  |
| __________________________________________ | |
